# Studying engagement in educational settings: a mapping review on high-impact academic engagement research

**DOI:** 10.3389/fpsyg.2025.1519509

**Published:** 2025-01-31

**Authors:** Paola Loyola-Carrillo, Alejandro Vega-Muñoz, Guido Salazar-Sepúlveda, Miseldra Gil-Marín, José Adsuar-Sala

**Affiliations:** ^1^Departamento de Matemáticas y Ciencias de la Ingeniería, Universidad Bernardo O’Higgins, Santiago, Chile; ^2^Centro de Investigación en Educación de Calidad para la Equidad, Universidad Central de Chile, Santiago, Chile; ^3^Facultad de Ciencias Empresariales, Universidad Arturo Prat, Iquique, Chile; ^4^Facultad de Ingeniería, Universidad Católica de la Santísima Concepción, Concepción, Chile; ^5^Facultad de Ingeniería y Negocios, Universidad de Las Américas, Concepción, Chile; ^6^Public Policy Observatory, Universidad Autónoma de Chile, Santiago, Chile; ^7^Faculty of Sport Sciences, University of Extremadura, Cáceres, Spain; ^8^Faculdade de Motricidade Humana, Universidade de Lisboa, Lisbon, Portugal

**Keywords:** engagement, educational research, behavioral studies, prolific author, h-index

## Abstract

**Introduction:**

Academic engagement provides opportunities and resources for students to engage in socio-educational interactions and learning. Our study provides an overview of high-impact research in academic engagement and the potential causes of its high valuation in the scientific community.

**Methods:**

We conducted a mapping review using bibliometric analysis of 1,607 articles indexed in Web of Science, processed mainly by VOSviewer software.

**Results:**

The publication of selected articles grew exponentially year by year, presenting concentration levels of 1% in authorship, 49% in a single country, and 5% in journals, identified as outstanding Keywords plus® central aspects of academic engagement (classroom-social environment and school engagement), in addition, 6% were in highly cited articles.

**Conclusion:**

These highly cited articles (6%) are associated with authors with high levels of publication. The most cited current topics relate to the motivation and emotional aspects of academic engagement.

## 1 Introduction

Engagement is a persistent, pervasive, positive, and satisfying affective-cognitive mental state with work, characterized by vigor, dedication, and absorption (Schaufeli et al., 2022), As an adaptation of engagement, academic engagement is reconceptualized to the academic and social environment of education, with characteristics that provide opportunities and resources for students to engage in academic learning and social interactions (Wang and Hofkens, 2020). Academic engagement with learning processes allows the optimization of academic performance and is an important construct for promoting interest, enjoyment, and psychological wellbeing among students (Medrano et al., 2015). Emotional engagement is essential for academic success and psychological wellbeing, influenced by the fulfillment of psychological needs, social relationships, and supportive environments. Autonomy, competence, and relatedness are key predictors, with studies showing that learning contexts supporting these needs enhance engagement (Park et al., 2012; Shih, 2008).

Thus, academic engagement is of interest to higher education institutions in relation to student dropout and the possibility of it being a significant predictor of early student dropout intentions (Truta et al., 2018). Similarly, Ketonen et al. (2016) identified four latent student profiles (engaged, disengaged, undecided, and alienated) according to engagement with the study: study-related burnout, lack of interest, lack of self-regulation, and uncertainty in career choice. Engaged students received the highest scores, while disengaged and undecided students scored the worst. This reinforces the conclusions of Casuso-Holgado et al. (2013) study on academic engagement and performance, in which, despite gender differences, the grade point average is the academic index most strongly associated with academic engagement. Social factors, including teacher-student relationships and peer support, play a pivotal role. Positive interactions with teachers and a caring school climate promote belonging and engagement, especially for minority and low SES students (Ulmanen et al., 2016; Krauss et al., 2022). One variable that does not appear innocuous for academic engagement is ethno-racial identity; fostering parental cultural socialization in relation to ethno-racial pride could promote academic engagement (Bakth et al., 2022).

It has also been shown that students who feel higher levels of psychological resources are more academically engaged, which has a positive impact on their academic performance (Martínez et al., 2019). Research also highlights the dynamic nature of engagement, where behavioral, cognitive, and emotional components influence each other over time (Hong et al., 2020). Overall, emotional engagement emerges from the interplay of psychological and social dimensions, with supportive environments fostering better academic and emotional outcomes (Park et al., 2012; Shih, 2008; Ulmanen et al., 2016; Krauss et al., 2022).

Academic motivation significantly influences student performance and engagement, with personal self-regulation and perceived parental and teacher support being key (De la Fuente et al., 2017; Simpkins et al., 2020). Motivational profiles, such as confidence and intrinsic motivation, predict academic adjustment (Linnenbrink-Garcia et al., 2018). Active methodologies, such as project-based learning, foster self-regulatory strategies, although they may decrease satisfaction (Galand et al., 2010). In STEM, gender inequalities in support and engagement persist (Patall et al., 2018). Learning goals help prevent disaffection (Valle et al., 2015), while motivational resilience and teacher support are essential for meeting challenges and balancing demands (Skinner et al., 2020; Guvenc, 2015).

Thus, positive emotions build psychological capital and academic engagement in students, thereby improving their academic performance (Carmona-Halty et al., 2021). In addition, fostering skills to understand and consider others’ perspectives, known as social perspective-taking (SPT), is crucial for students’ academic and social development (Kim et al., 2018). According to the temporal perspective theory, how people value the past, present, and future influences their actions. Considering students, only the future temporal perspective (the importance that people place on the future) uniquely predicted academic engagement intention and academic performance (Barnett et al., 2020). Thus, it is key to reinforce students’ psychological security, which is understood as a mental state of feeling safe and supported in the educational environment, because it is a positive predictor of academic performance (Tatiana et al., 2022). In addition, students with high emotional self-efficacy–that is, those who were confident in their ability to manage emotions in the context of learning in a digital society–obtained better academic results (Yu et al., 2022).

Regarding the measurement of academic engagement, although the Utrecht Work Engagement Scale and its three factors: vigor, dedication, and absorption are commonly used (Schaufeli et al., 2022; Barragan-Martin et al., 2021; Ng et al., 2022; Rodríguez-González et al., 2023). By contrast, the study by Wefald and Downey (2009) did not confirm a trifactorial structure and found that engagement and satisfaction were closely related constructs. Thus, the discrepancies in the number and nature of the dimensions that make up academic engagement (Tomas et al., 2022) give openness to other psychometric measurement instruments, such as the University Student Engagement Inventory, (USEI), also trifactorial: (behavioral, emotional, and cognitive) (Assunçao et al., 2020; Sinval et al., 2021; Freiberg-Hoffmann et al., 2022) and the relationship of academic engagement with a number of other constructs: stress and burnout (Gómez et al., 2015), positive emotions, autonomy and self-efficacy (Oriol-Granado et al., 2017), teacher work engagement (Zhang and Yang, 2021), satisfaction and frustration (Buzzai et al., 2021), and self-esteem and motivation (Acosta-Gonzaga, 2023).

Previous bibliometric analyses on academic engagement define it as the interaction between academics and non-academic actors, such as industry, to promote knowledge exchange, cooperation, and the application of research to address societal and technological challenges (Pham et al., 2024; Nast et al., 2025), for example, Abramo and D'Angelo (2022) examine university-industry collaboration in Italy, focusing on factors influencing academics’ willingness to engage. Pham et al. (2024) review the evolution of academic engagement, highlighting technology transfer as a key issue. Lastly, Nast et al. (2025) explore the link between scientists’ interactions with non-academic actors and high-impact research in Spain, finding that renowned scientists are best positioned to leverage these opportunities. But none of these studies focus specifically on the educational settings.

Therefore, in contrast to previous publications (Abramo and D'Angelo, 2022; Pham et al., 2024; Nast et al., 2025), our study aims to provide a worldwide panoramic view of academic engagement research and identify high-impact research on this type of behavior in educational settings. It answers how certain variables are related to high citation counts, depending on the age of the documents, characteristics of their authors, national authorship ascriptions, journals of publication, and open access to these documents. Asking the question, “What are the highest impact mainstream research publications on academic engagement in educational settings?” allows us to identify global research and training benchmarks, providing input to the epistemic community of researchers and educational decision-makers.

## 2 Methods

Based on a dataset extracted from the Core Collection of Web of Science (WoSCC) on July 15, 2024 (Clarivate, 2023), with the thematic search vector of Academic Engagement [TS = (academic NEAR/0 engagement)], refined by the Web of Science Index: Social Sciences Citation Index (SSCI) or Science Citation Index Expanded (SCI-EXPANDED, SCIE), unrestricted thematically and temporarily. The research process develops a cartographic review, and therefore seeks to characterize the quantity and quality of the literature and other key characteristics, identifying research needs (Grant and Booth, 2009). Using Web of Science-WoS articles (Clarivate, 2023) as a reference, given their recognized quality among researchers worldwide (Serrano et al., 2019). The authors selected the SSCI-WoSCC and SCIE-WoSCC databases because with respect to Scopus, the journals indexed in both WoS databases present a high duplicity of indexing in Scopus. However, Scopus journals, which do not present a double indexing with the SSCI and SCIE bases, have not been considered because “Scopus covers a higher number of journals, but with lower impact (average citations) and limited to recent articles” (Chadegani et al., 2013, p. 24). For the identical reasons we have not considered the Emerging Sources Citation Index (ESCI) of WoSCC. The length of the bases is relevant, Scopus is only 20 years old as a commercial product of Elsevier and ESCI-WoS, is a base created in 2015, which affects the historical citation counts for subsampling (e.g., h-index), given the longitudinal nature of the mapping reviews. Consequently, the analytical procedure of the present study preferred impact to the number of journals. As a document type registered in WoS, we have considered only articles, given that according to Cambridge University Press (2024) they are the most common type of article in the world of periodicals, contain pieces of original research that contribute directly to their field, apply to all disciplines, and are written by experts, for experts, and must meet the highest standards of peer review and scholarly communication. Articles are written by experts, for experts, and must meet the highest standards of peer review and scholarly communication.

The thematic search tag TS performs a simultaneous search on the following fields: title, keywords, author, abstract, and Keywords Plus® and the word proximity operator (NEAR) and simultaneously incorporates both words (Clarivate, 2023). Then, based on the “Guidelines for advancing theory and practice through bibliometric research” (Mukherjee et al., 2022), both performance analysis and science mapping were performed. For performance analysis, the bibliometric laws (Haddow, 2018) of Price (1976), Lotka (1926), and Zipf (1932), and Hirsch (2005) index were used, while science mapping focused on co-authorship analysis using VOSviewer software for co-authorship and co-occurrence analysis, discovering the social relationships of authors, organizations, or countries and thematic relationships between keywords (Van Eck and Waltman, 2010). The independent subsamples for the documents included in each type of analysis are detailed in Table 1. Only the Keywords plus© are sampled in a dependent manner based on the articles selected by the Hirsch index (h-index).

1. Price’s Law allows the analysis of the exponential growth of science (exponential growth adjustment of the annual publication number) as an expression of the critical mass of knowledge that is interesting to study (Price, 1976; Dobrov et al., 1979).
2. Lotka’s Law allows the segregation of authors of high production in a specific subject from those who have an ephemeral step in a particular area of scientific knowledge (a high percentage of authors who only present one or a relatively small number of published papers). To estimate the concentration of authors, the square root is applied to the total number of authors, which is then adjusted according to a discrete number of publications, and the resulting set of authors are called prolific authors (Lotka, 1926; Nicholls, 1988; Tsai, 2013).
3. Bradford’s Law concentrates on journals, mainly in what is known as Bradford’s core, the smallest subset of journals that manage to concentrate on one-third of the total number of documents studied. The subsets that manage to concentrate on the remaining documents according to their increasing order in the number of journals are known as Zones 1 and 2. However, attention has been focused on the Bradford core as a production environment that tends to congregate the most specialized authors, reviewers, and editors in a specific topic of study (Bulik, 1978; Desai et al., 2018).
4. The Hirsch index determines the relative impact of scientific productivity on a corpus of selected articles. It is expressed as the value n of documents, implying that these n documents have obtained n or more citations on a common counting basis for all these (Hirsch, 2005; Crespo and Simoes, 2019). In addition, we studied the relationship between the age of publication and the number of citations and the inclusion of an article in the h-index in relation to: (1) the authorship of one or more prolific authors, (2) the affiliation of one or more authors to a prolific country, (3) publication in a journal specialized in the subject (belonging to the Bradford core), or (4) some form of open access to the article.
5. Regarding these last four items, a nonparametric descriptive statistical analysis was used with the SPSS program. Using the nonparametric Chi-square correlation coefficient (χ2), whose correlation is significant for a *p*-value at the 0.05 level (ideally 0.01), a case in which a degree of association between two variables is statistically evident (Romero Suárez, 2012; Molina-Arias, 2017).

**Table 1:** Characterization of bibliometric subsampling.

Phase	Variable	Value (or sample, *n*)	Unit	Subsampling criteria for inclusion	Subsample included	Documents included	Inclusion rate
1	Time	1982–2024	Year	Period without blanks	1992–2023 years	1478	0.92
2	Authors	4,667	Person	Lotka’s Law	59 authors*	303	0.19
3	Place (Affiliation)	87	Country /Territory	Prolific authors lower limit	40 countries**	1566	0.97
4	Journals	522	Journal	Bradford’s Law	24 journals	519	0.32
5	Documents	1,607	Article	Hirsch’s index (h-index)	92 documents	92	0.06
6	Keywords Plus	2,649	Words	Zipf’s Law	35 words	92	0.06

*Database field: author full names, **database field: addresses.

Zipf’s Law refers to the concentration of word usage in the language. Here, the keywords assigned as metadata by WoS or Keywords plus© were used as a basis to study this concentration, highlighting the most used keywords in the set of articles, using the square root over the set of keywords as an estimate. This was then adjusted according to a discrete number of keywords. The resulting set of Keywords plus© is known as outstanding keyword plus (Zipf, 1932; Merediz-Solà and Bariviera, 2019) (Table 1).

## 3 Results

### 3.1 Results of scientific production on academic engagement

**Figure 1:**
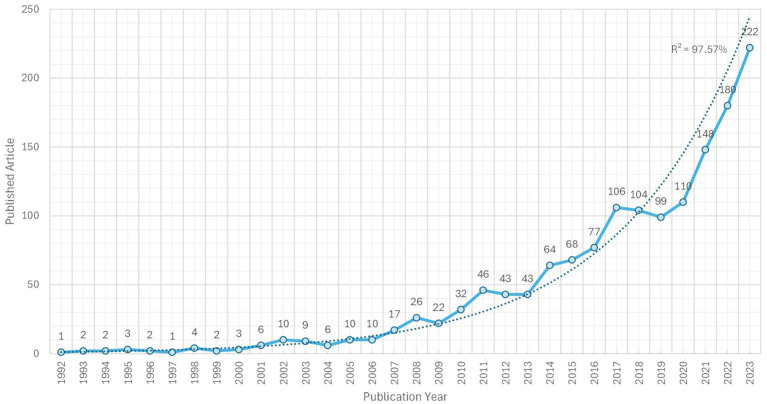
Time series and trend of publications on academic engagement. Blue line is time series and dotted lines is an exponential trend.

The 1,607 articles extracted from the WoS Core Collection cover the period 1982–2024; however, only present a continuous full-year record (without years with blank data) between 1992 and 2023. For this period, including 1478 documents, where it is possible to analyze possible exponential growth, R^2^ was 98%. Thus, according to Price (1976)’s Law, scientific production shows a critical mass of interesting knowledge to be studied. Regarding the research areas - WoS, articles related to 159 research areas are collected, and although a journal (and consequently its articles) can be indexed to several areas simultaneously, we indicate that 840 of 1,607 articles are indexed to the WoS area of Psychology (52%), and 542 of 1,607 articles to the area of Education and Educational Research (34%), adding between both categories, discounting duplicates, 1,240 of 1,607 articles (77%) (Figure 1).

**Figure 2:**
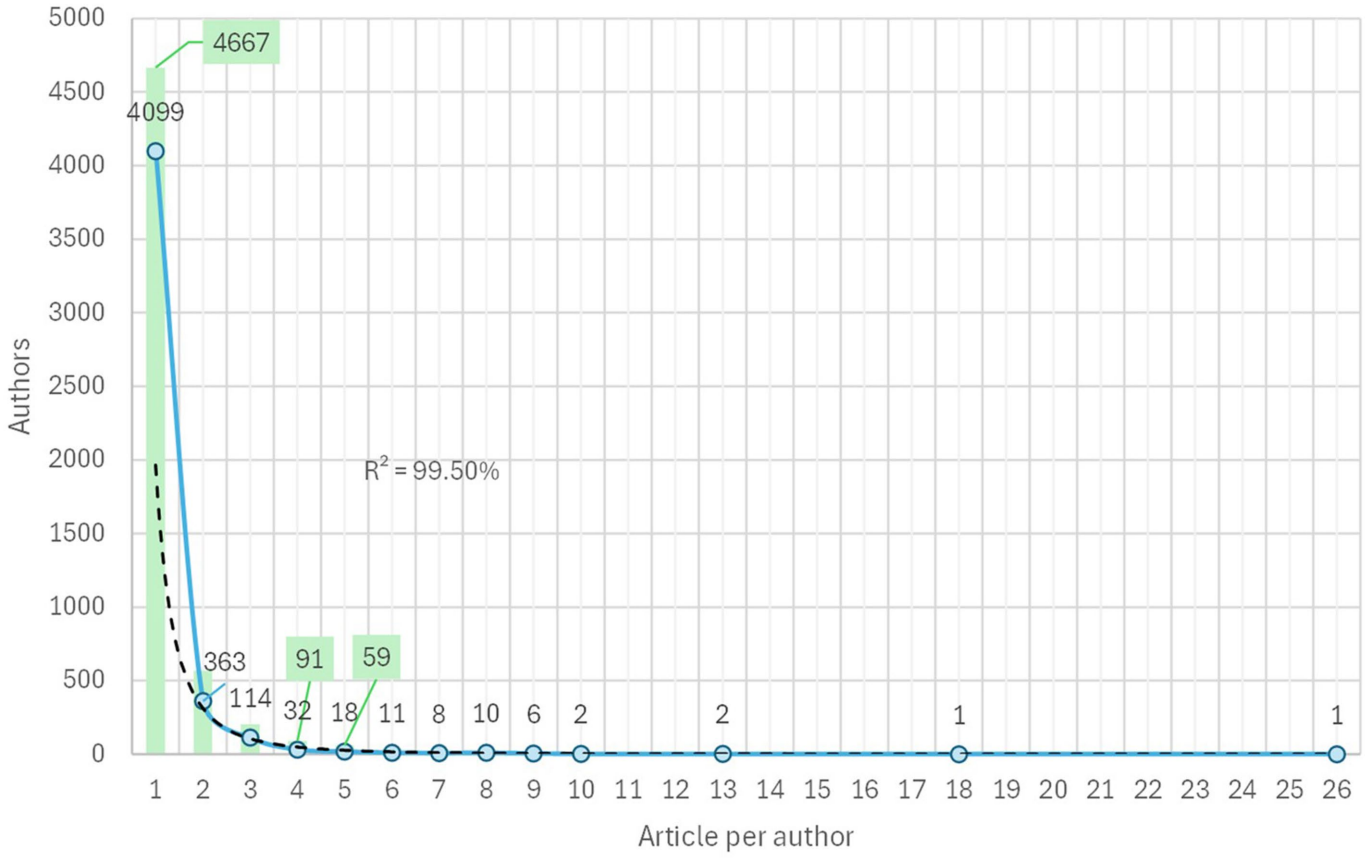
Relationship between authorship and the scientific production level. Blue line is time series and dotted lines is a power fit trend.

This number of articles generated was the work product of 4,667 researchers, but of these authors 4,099 only contributed one article. Thus, Figure 2 shows the contribution levels of these 4,667 authors from 1 to 26 articles with a power fit of 99.5%; according to Lotka (1926)’s Law, the number of prolific authors can be estimated at 68 authors [SQRT (4667) = 68].

**Figure 3:**
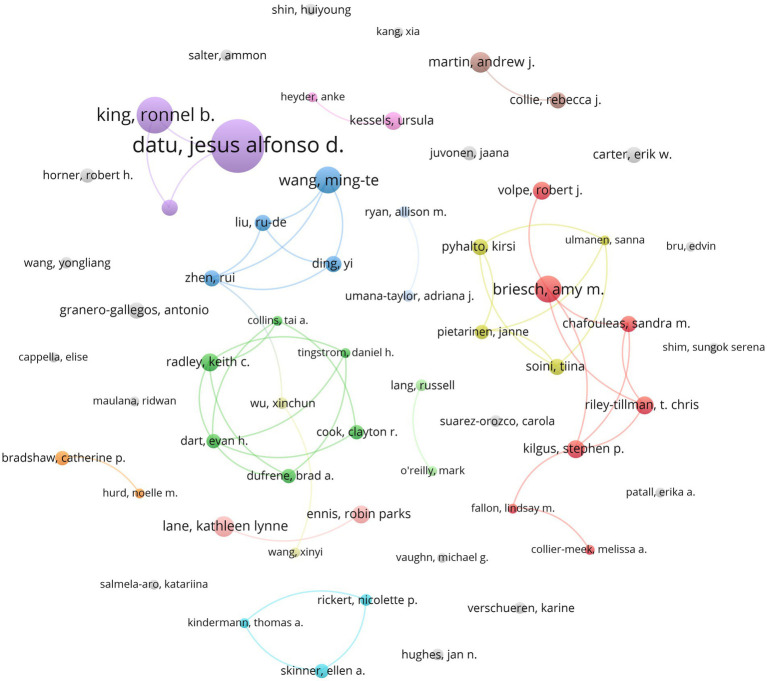
Prolific co-authorship graph (colors indicate same author cluster).

From Figure 2, it can be observed that 91 authors had four or more published articles, and 59 authors had five or more published articles and academic engagement; therefore, prolific authors were estimated at 59, equivalent to inclusion of 303 documents (without duplicates). This small group of authors with a high level of production in the subject (five or more articles) maintained co-authorship relationships, as shown in Figure 3. The most prolific author was Dr. Jesús Alfonso D. Datu, an academic from the Faculty of Education, University of Hong Kong, ORCID: https://orcid.org/0000-0002-8790-1113.

Figure 3 shows that 41 of the 59 authors were grouped into co-authorship teams, with 13 clusters at this level of scientific production, including two triads and seven dyads. All nodes in grey are authors who can be considered solitary authors at this level of production (Table 2).

**Table 2:** Prolific author clusters and national affiliations.

Cluster	Co-authors (articles)	*N*	Countries of authors
1	Briesch (13), Chafouleas (8), Collier-Meek (5), Fallon (5), Kilgus (9), Riley-Tillman (9), Volpe (9).	7	USA
2	Collins (5), Cook (7), Dart (7), Dufrene (7), Radley (9), Tingstrom (5).	6	USA
3	Ding Y (8), Liu RD (8), Wang MT (13), Zhen (8).	4	China, USA
4	Pietarinen (7), Pyhältö (8), Soini (8), Ulmanen (5).	4	Finland
5	Datu (26), King (18), Valdez (8).	3	China (HK), Philippines
6	Kindermann (5), Rickert (6), Skinner (7).	3	USA
7	Bradshaw (7), Hurd (5).	2	USA
8	Collie (8), Martin (10).	2	Australia
9	Heyder (5), Kessels (9).	2	Germany
10	Ennis (9), Lane (10).	2	USA
11	Lang (6), O’Reilly (5).	2	USA
12	Ryan (6), Umaña-Taylor (6).	2	USA
13	Wang X (5), Wu (6).	2	China

**Figure 4:**
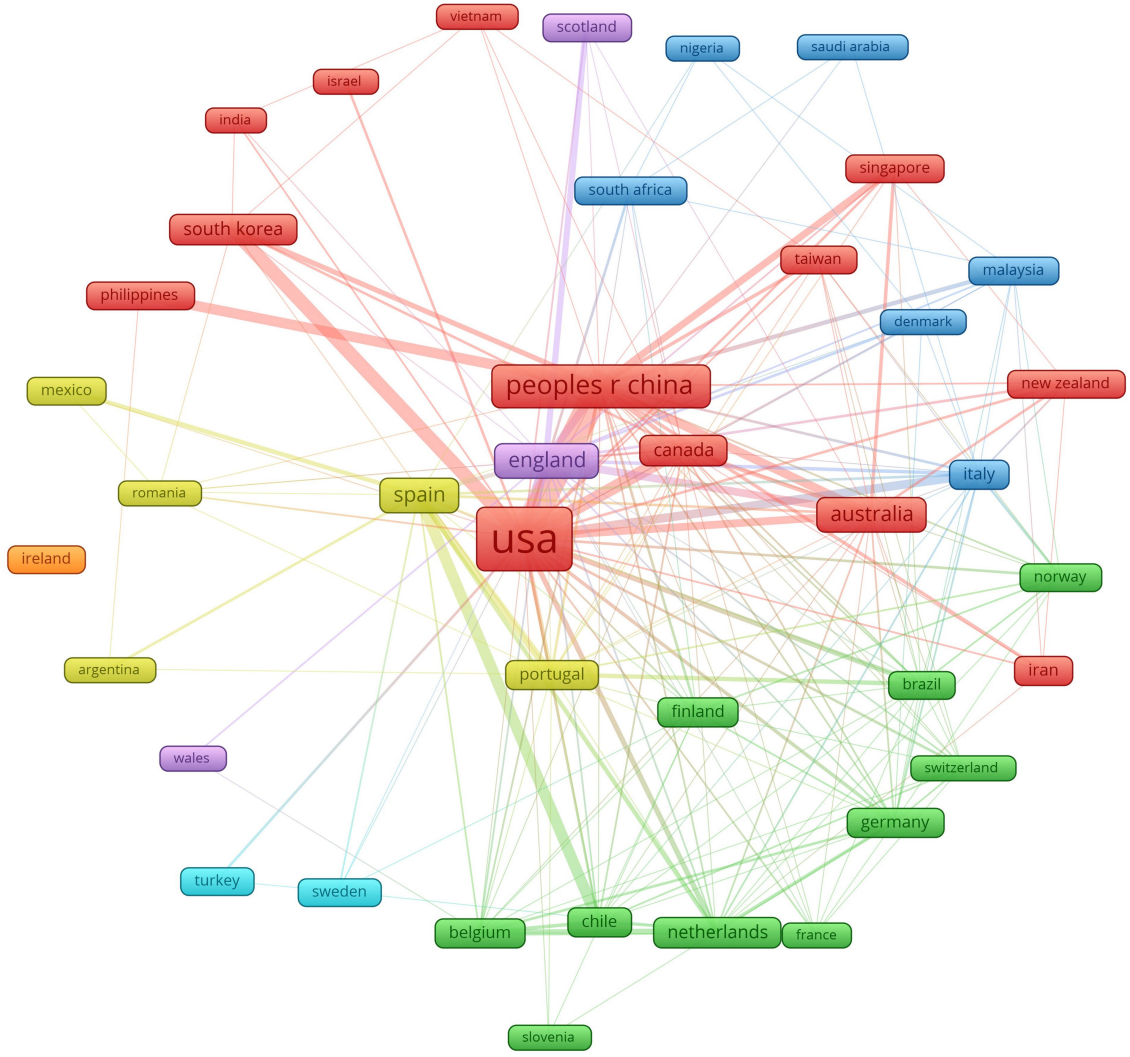
National co-authorship graph (colors indicate same author cluster).

Under the same level of stringency (five or more published articles), Figure 4 shows the co-authorship at the country level (40 countries). The sizes of the frames represent the volume of production, the arcs represent the co-authorship relationships between countries, and the seven colors divide the countries by their degree of association in terms of co-authorship: red (13 countries), green (10 countries), blue (six countries), yellow (five countries), violet (triad), light blue (dyad), and orange (one country only). The levels of contribution to world knowledge production in academic engagement in the USA (red, 788 articles), China (red, 239 articles), and Spain (yellow, 108 articles) stand out, as well as the strong relationship between the two (red edges) (Table 3).

**Table 3:** Prolific countries according to their contribution to scientific production.

Prolific country	Articles	Citations	Citations per article	% Contribution at 1607
USA	788	31746	40	49.0%
China	239	3865	16	14.9%
Spain	108	1943	18	6.7%
Australia	98	2260	23	6.1%
England	94	3131	33	5.8%

Finally, in relation to the scientific production of academic engagement, it is necessary to note that 24 out of 522 journals accounted for approximately one-third of the 1,607 articles published (519 articles) on this subject between 1982 and 2024 (Table 4).

**Table 4:** Bradford nucleus journals and their web of science impact characteristics.

Journal on nucleus of Bradford	Art.	JIF–WoS (2023)	Best Qx (2023)
Frontiers in Psychology	70	2.6	Q2
Journal of School Psychology	37	3.8	Q1
Journal of Youth and Adolescence	31	3.7	Q1
Psychology in The Schools	30	1.8	Q3
Current Psychology	30	2.5	Q2
Journal of Positive Behavior Interventions	29	1.4	Q3
Educational Psychology	23	3.6	Q1
Learning and Individual Differences	22	3.8	Q1
Behavioral Disorders	22	2.1	Q1
Studies in Higher Education	21	3.7	Q1
Social Psychology of Education	20	3.2	Q1
Children and Youth Services Review	19	2.4	Q1
School Psychology Review	16	3.9	Q1
Journal of Educational Psychology	15	5.6	Q1
Sustainability	15	3.3	Q2
Journal of Applied Developmental Psychology	15	2.2	Q2
Education and Information Technologies	15	4.8	Q1
Contemporary Educational Psychology	14	3.9	Q1
Developmental Psychology	14	3.1	Q2
Journal of Technology Transfer	13	4.6	Q1
Revista de Psicodidactica	12	3.8	Q1
Plos One	12	2.9	Q1
International Journal of Environmental Research and Public Health	12	N.A.	N.A.
Journal of Adolescence	12	3.0	Q2

### 3.2 Scientific production impact on academic engagement

**Figure 5:**
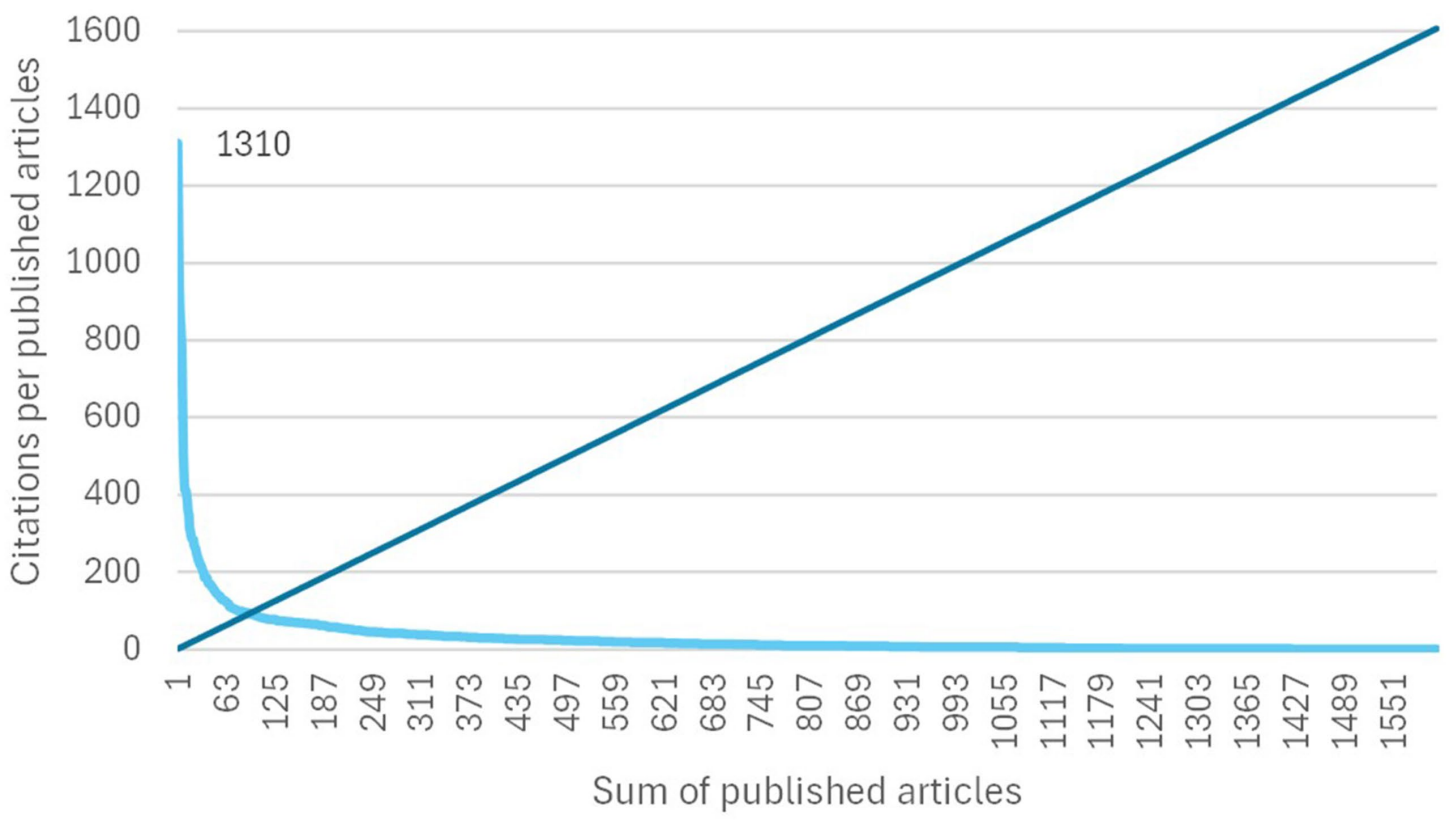
h-Index estimation. Light blue line is time series and blue line is a counting of articles.

With reference to the impact of scientific production, it was possible to determine a subset of 92 articles (5.7%) according to the Hirsch index (h-index), represented by the intercept shown in Figure 5. One article by Furrer and Skinner (2003) stands out, with 1,310 citations in the WoS Core Collection on the date of data extraction.

**Figure 6:**
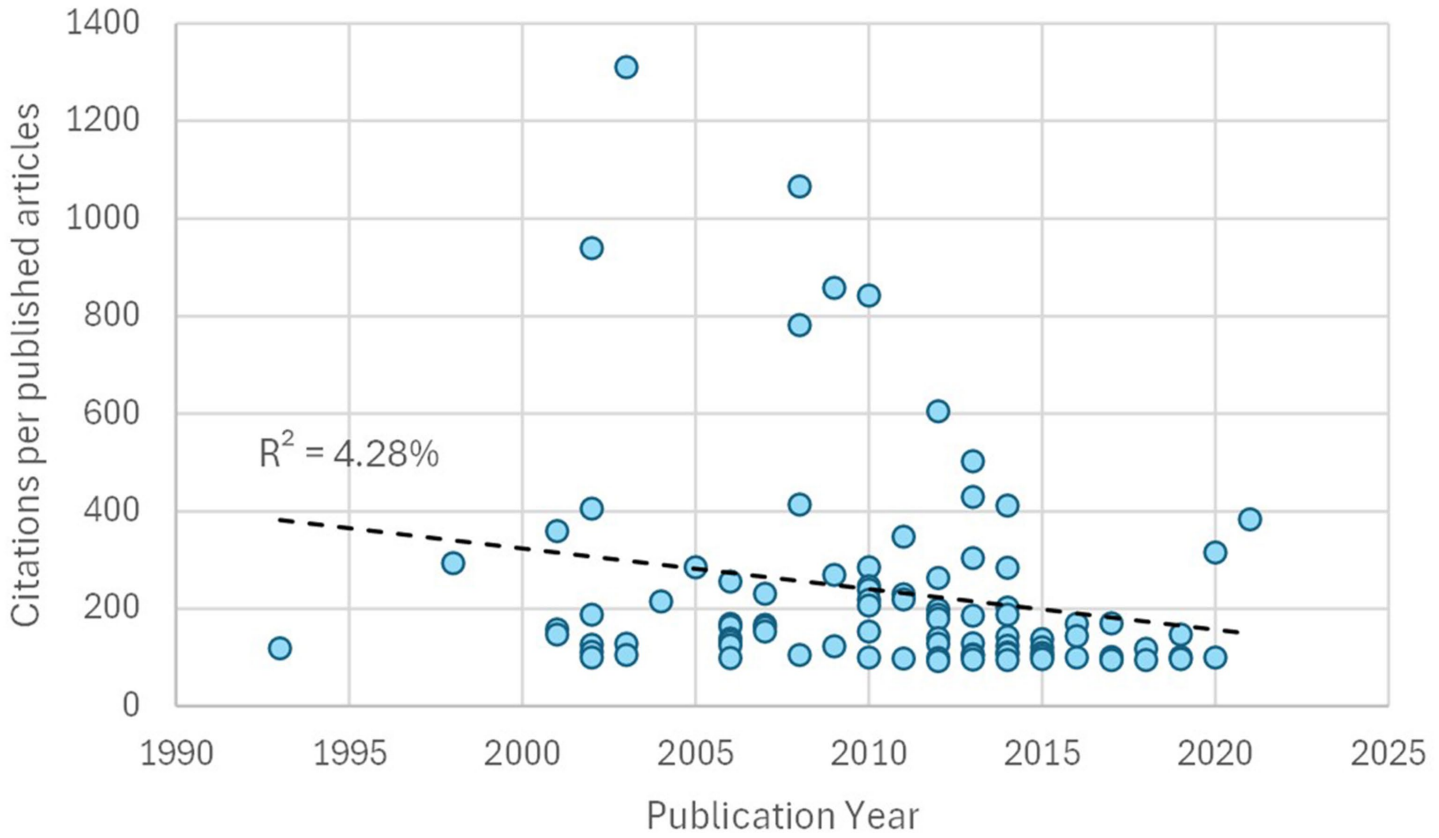
Time of publication and citations of articles in the h-index.

In search of an explanation for this high number of citations, a relationship was established between the year of publication and the number of citations within these 92 publications. As shown in Figure 6, the percentage of adjustment (R^2^) was <5%; therefore, we can conclude that the volume of citations received was not dependent on the age of publication of the document. In addition to the article by Furrer and Skinner (2003) (1,310 citations), another paper by Skinner et al. (2008) stood out, exceeding 1,000 citations.

We also explored the possible associations between the inclusion of an article in the h-index with: (1) authorship by one or more prolific authors, (2) affiliation of one or more authors with a prolific country, (3) publication in a journal specializing in the subject (belonging to the Bradford nucleus), or (4) some form of open access to the article. The relationships with the degrees of association are reported in Table 5.

**Table 5:** Relationship between h-index articles and other variables.

Variables	Asymptotic significance (2-sided)	Degree of freedom	Significant relationship between variables
Prolific authors	6.948	1	0.008**
Prolific country	1.541	1	0.214
Journal on nucleus of Bradford	0.969	1	0.325
Open access article	1.591	1	0.207

*p ≤ 0.050, **p ≤ 0.010.

**Figure 7:**
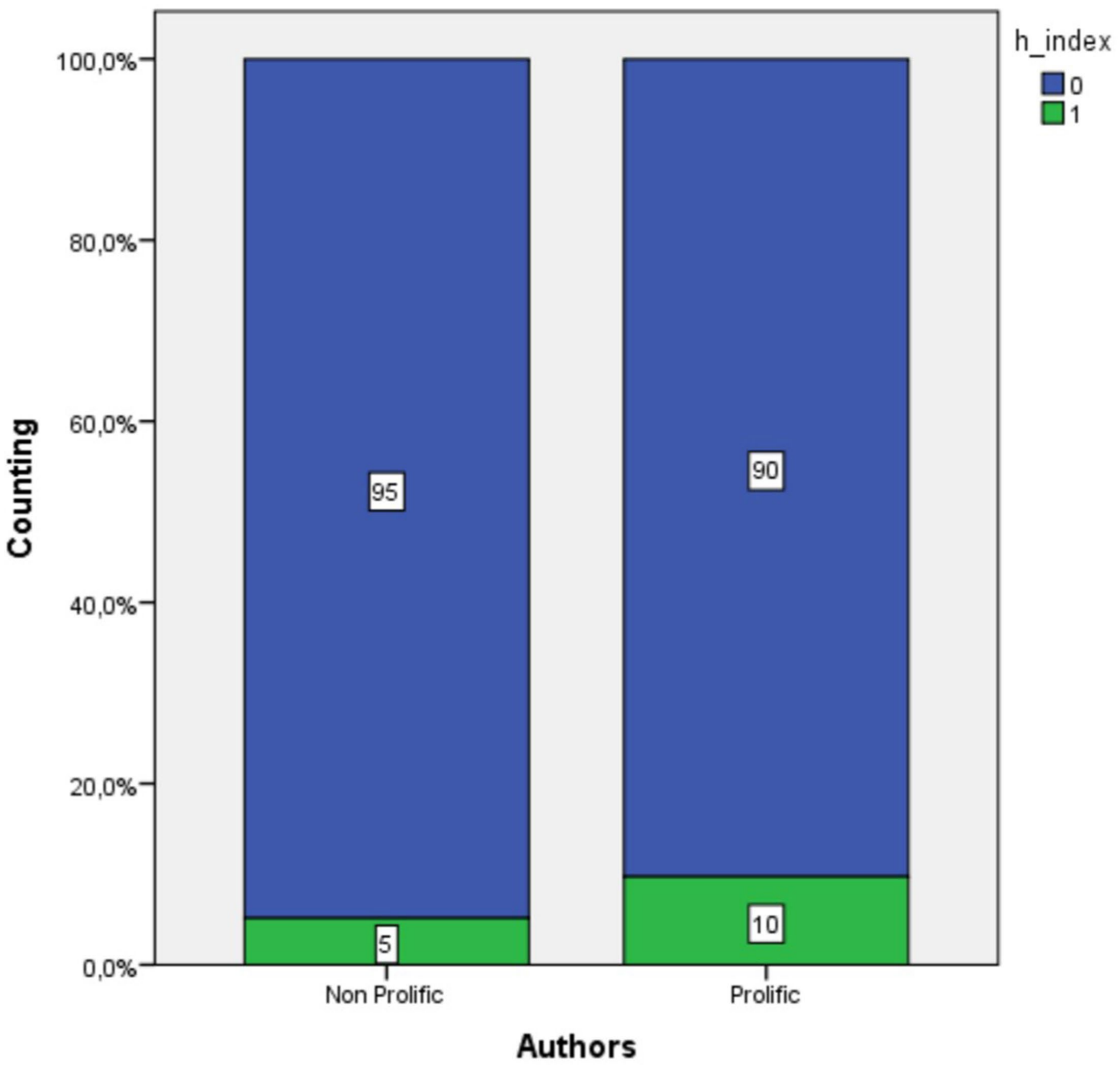
Relation between the proliferation level of authors and h-index inclusion.

There was no evidence to determine the degree of association between the articles included in the h-index and the affiliation of one or more authors to a prolific country, publication in a journal specializing in the subject, or any form of open access to the article. In contrast, there was evidence of an association between an article in the h-index and authorship by one or more prolific authors. Figure 7 shows that the percentage of articles in the h-index doubled from 5 to 10% when they were self-authored by one or more prolific authors. Thus, this result affirms that, for the set of 1,607 articles on academic engagement under study, the only relevant variable for high citation is that the article is by a prolific author in this topic.

**Figure 8:**
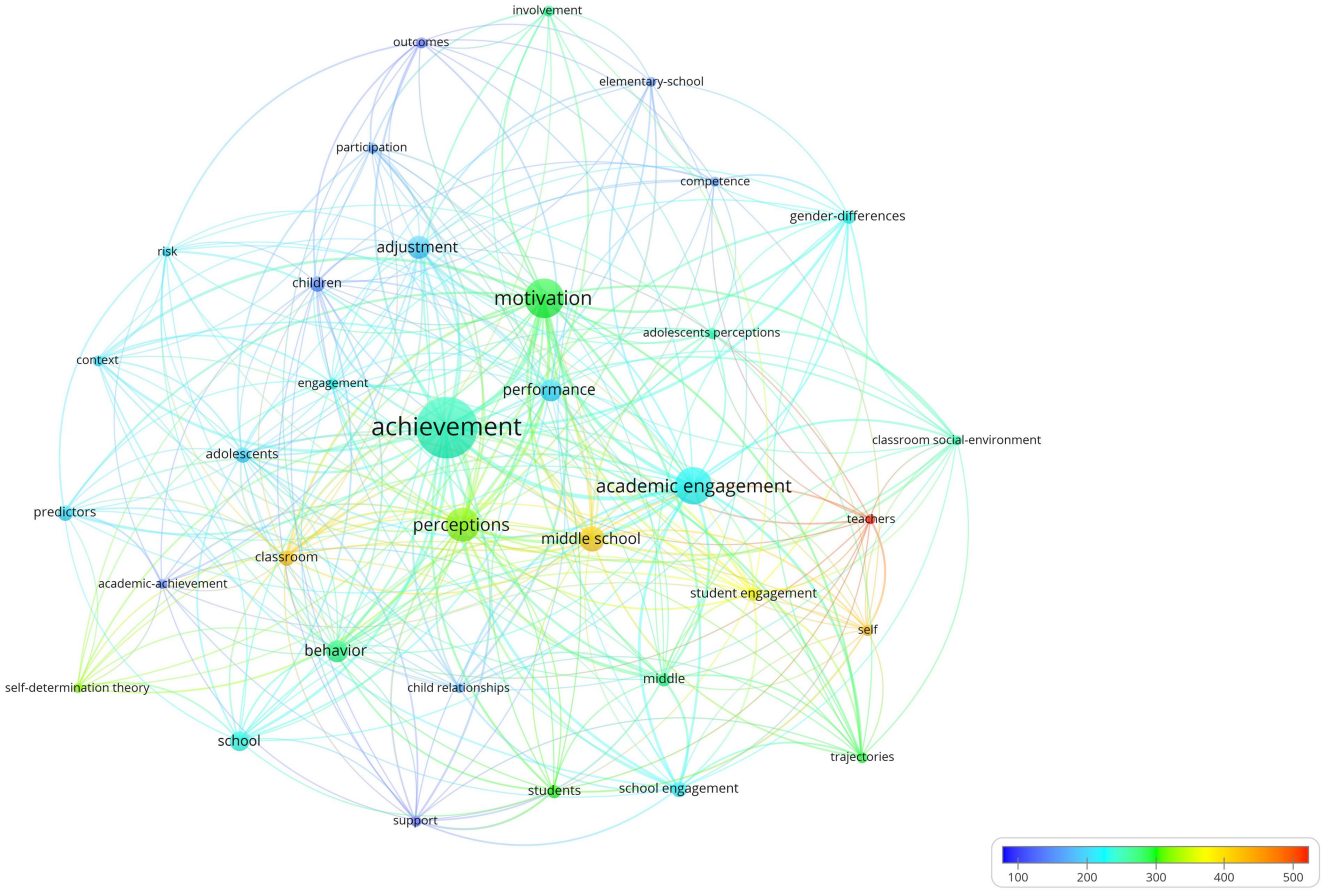
Keywords plus co-occurrence graph: colors indicate average citations.

Another interesting finding is the most cited topics associated with these 92 articles, which are represented based on the 35 Keywords plus® with the highest number of occurrences. The average number of citations is shown in Figure 8. Identifying only four keywords plus® of very high relevance in academic engagement studies:

- Teachers with 530 average citations in 5 h-index articles: Skinner et al. (2008), Skinner et al. (2009), Wang and Eccles (2013), Lee and Smith (1993), and Hughes and Coplan (2010),
- Self with 424 average citations in six articles stand out: Skinner et al. (2008), Skinner et al. (2009), Hughes and Coplan (2010), Middleton (2010), Datu et al. (2016), and Pietarinen et al. (2014),
- Classroom with 411 average citations in 8 h-index articles: Furrer and Skinner (2003), Jang et al. (2010), Farmer et al. (2011), Gasiewski et al. (2012), Fall and Roberts (2012), Wentzel and Watkins (2002), Tucker et al. (2002), and Engels et al. (2016), and
- Middle school with 402 average citations in 13 h-index articles: Furrer and Skinner (2003), Skinner et al. (2008), Wang and Eccles (2013), Pietarinen et al. (2014), Farmer et al. (2011), Wang and Degol (2013), Johnson et al. (2001), Wang and Sheikh-Khalil (2014), Wang and Degol (2013), Johnson et al. (2001), Wang and Sheikh-Khalil (2014), Anderson et al. (2004), Schwartz et al. (2006), Suárez-Orozco et al. (2010), Zimmer-Gembeck et al. (2006), and Liu et al. (2018).

The finding of these four relevant keywords (Teachers, Self, Classroom, Middle school) will be complemented below with a view of contemporaneity.

**Figure 9:**
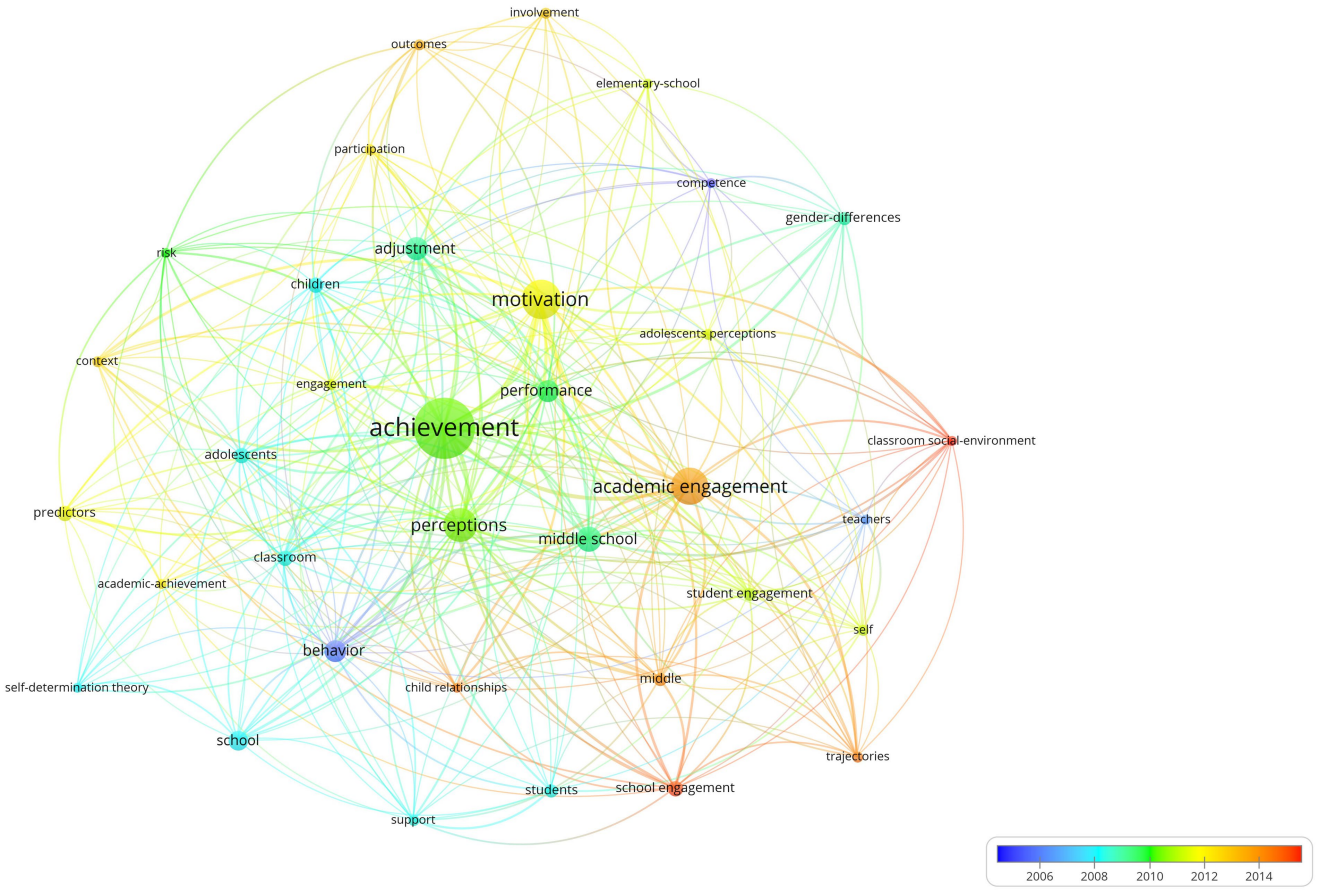
Keywords plus co-occurrence graph: colors indicate average publication years.

Additionally, in Figure 9, the Keywords plus® with more recent average dates were:

- Classroom social-environment with 2015.00 average publication year in 5 h-index articles: Wang and Eccles (2013), Pietarinen et al. (2014), Wang and Degol (2013), Liu et al. (2018), and Strati et al. (2017),
- School engagement with 2014.62 average publication year in 8 h-index articles: Skinner et al. (2009), Engels et al. (2016), Liu et al. (2018), Wang and Degol (2014), Hospel and Galand (2016), Wang and Huguley (2012), Rimm-Kaufman et al. (2015), and Vollet et al. (2017).

Thus, the six articles that intersected the highest number of citations and topical issues were as follows: Skinner et al. (2009), Wang and Degol (2013), Pietarinen et al. (2014), Engels et al. (2016), Wang and Eccles (2013), and Liu et al. (2018), among which we identified the importance of motivation (Skinner et al., 2009; Wang and Degol (2013); Wang and Eccles, 2013), and emotion (Pietarinen et al., 2014; Liu et al., 2018) aspects (Table 6).

**Table 6:** Relevant contemporaneous topics in h-index articles and other variables.

Studied topic	Motivation			Emotion	
Article	Skinner et al. (2009)	Wang and Eccles (2013)	Wang and Degol (2013)	Liu et al. (2018)	Pietarinen et al. (2014)
Prolific author clusters	6	3	3	3	4
Prolific authors (Articles)	Skinner (7), Kindermann (5)	Wang MT (13)	Wang MT (13)	Ding Y (8), Liu RD (8), Zhen (8)	Pyhältö (8), Soini (8), Pietarinen (7)
Prolific country	USA	USA	USA	China, USA	No
Journal on nucleus of Bradford	No	No	No	Educ. Psychol.	No
Open access	No	No	Green Accepted	No	No
Web of science index	SSCI, SCIE	SSCI	SSCI	SSCI	SSCI
Times cited, WoS Core*	857	502	429	117	142

*Extraction data: 15 July 2024.

## 4 Discussion

From a methodological viewpoint, our mapping review uses fundamental bibliometric laws (Haddow, 2018). Thus, when presenting the temporal evolution of the sample of selected articles, unlike the bibliometric studies on behavior in the educational setting by López-Belmonte et al. (2021), Alcaraz-Garcia (2021), and García-Chitiva (2021), our work uses Price’s Law (1976) to account for the implications of the exponential growth of science. This set of selected articles was obtained from WoS, as well as other bibliometric research on behavioral studies in education (López-Belmonte et al., 2021; Baek and Doleck, 2022; Tiberius and Weyland, 2023). The data was analyzed using VOSviewer (Van Eck and Waltman, 2010), a software commonly used by other similar studies (Tiberius and Weyland, 2023; Dong and Zeb, 2022; Ling et al., 2023).

Regarding our results, (1) the estimation of nucleus journals using Bradford’s Law (Bulik, 1978) allowed us to identify Frontiers in Psychology, International Journal of Environmental Research and Public Health, Plos One, Sustainability, as well as Tiberius and Weyland (2023) and Dong and Zeb (2022) as relevant journals; (2) the estimation of prolific authors led us to consider 1.3% of our total authors (4,667) with five or more published articles, which is comparable to the 1.5% of the total authors (1,505) with four or more published articles used by Tiberius and Weyland (2023); (3) in terms of territorial concentration, our results agree with Baek and Doleck (2022) for USA, China, and Spain, and with Dong and Zeb (2022) for USA, China, Australia, and United Kingdom (England); (4) unlike other articles that report prolific authors and results based on h-index (López-Belmonte et al., 2021; Dong and Zeb, 2022; Ling et al., 2023), our work also identifies the correlation between both sets by means of a chi-square test.

In contrast with other mapping review using bibliometrics on this topic (Pham et al., 2024), we attribute this to the neutrality of our search vector that our results identify as outstanding Keywords plus® central aspects of academic engagement (classroom social environment and school engagement), and the most relevant articles highlight the importance of motivation (Skinner et al., 2009; Wang and Eccles, 2013; Wang and Degol, 2013), and emotion (Pietarinen et al., 2014; Wentzel and Watkins, 2002) aspects. Our results also establish a distance compared to the concept of student engagement, which is usually understood as proximate. Torres-Castro (2024), as in our study, identifies a relevant role in the scientific production of USA, UK, Australia, China and Spain, and highlights psychological and behavioral perspectives as relevant categories of study. Along the same lines, with considerable precision and even greater proximity, our study resembles its findings with two of the main research themes of Aparicio et al. (2021): “Academic, social and personal involvement and environment,” and “Feelings and perspectives,” achieving convergence of both theoretical constructs in these topics.

## 5 Conclusion

This bibliometric mapping review on high-impact research in academic engagement concludes that the scientific production of researchers has grown at an exponential rate (R^2^ = 98%). This is a product of the contribution of 1,607 authors from 87 countries, with a co-authorship from the USA of 49%. However, according to Lotka’s law, of the total number of authors, only 59 were estimated as prolific (1.3%), contributing five or more publications on the topic studied, forming 13 co-authorship clusters (including triads and dyads), and highlighting the production level of a researcher affiliated with The University of Hong Kong. Bradford’s law identified 24 of 522 journals (4.6%), accounting for one-third of the published articles. The journal with the highest concentration was Frontiers in Psychology, with 70 papers indexed in the WoS Psychology, Multidisciplinary (JIF-Q2) category.

Regarding the impact of scientific production on academic engagement, the h-index, as a citation impact weighting factor, determined that 92 of 1,607 articles (5.7%) were relevant within the set of articles studied. The citation levels of these 92 articles did not depend on variables such as the age of publication, affiliation of one or more authors with a prolific country, publication in a journal specializing in the subject (belonging to the Bradford core), or having some form of open access to the article. However, this was associated with authorship by one or more prolific authors, as in the recent work of Nast et al. (2025), but in attention to another optic of academic engagement. A clear manifestation of the “Matthew effect” in science, which gives greater visibility and credit to high-profile scientists (Merton, 1968; Teixeira da Silva, 2021).

In addition, the outstanding Keywords plus® of the articles in the h-index, show that the cross between the highest average citations and the most current average years of citation, as relevant topics in the study of academic engagement, are the motivation and emotion aspects.

Knowing which are the highest impact research publications on academic engagement allows us to identify two topics of contemporary relevance in the study of academic engagement, motivation and emotion, as a convergence of five articles of the highest worldwide citation in this field of study. Articles that distinguish themselves thanks to our work as world reference documents for the epistemic community of academic engagement researchers to put them at the center of their studies, and for educational decision makers to see motivational and emotional aspects as relevant knowledge in the teaching of academic engagement.

Finally, as a future line of research, we recommend further empirical investigation of the aspects of academic engagement and their relationship with achievement, motivation, and emotion aspects. Thus, essential questions for future research could be:

- How does academic engagement influence student achievement?
- What emotional factors affect the relationship between academic engagement and achievement?
- How does intrinsic and extrinsic motivation impact academic engagement and performance?
- How does social support (family, peer, Teacher) modulate students’ engagement and emotions?
- What differences exist in academic engagement, achievement and emotions according to cultural or socioeconomic context?
- How does the type of task (individual or group) influence students’ engagement and emotions?
- What role do self-compassion and self-criticism play in academic engagement and its relationship with achievement?
- How does academic engagement affect students’ long-term resilience and motivation?
- How do educational technologies impact students’ academic engagement and emotions?
- What differences exist in academic engagement, motivation and emotions between different educational levels?

This question set provides a broad overview of how various factors influence academic engagement, emotions, and achievement, underscoring the importance of considering the diverse psychological, social, and cultural elements that shape students’ learning experiences.

## Data Availability

The original contributions presented in the study are included in the article/Supplementary material, further inquiries can be directed to the corresponding author.
